# Radial peripapillary vessel density as early biomarker in preperimetric glaucoma and amnestic mild cognitive impairment

**DOI:** 10.1007/s00417-022-05561-5

**Published:** 2022-01-22

**Authors:** Daniela Montorio, Chiara Criscuolo, Maria Angelica Breve, Roberta Lanzillo, Elena Salvatore, Vincenzo Brescia Morra, Gilda Cennamo

**Affiliations:** 1 Department of Neurosciences, Reproductive Sciences and Dentistry, University of Naples Federico II, Naples, Italy; 2 Eye Clinic, Department of Public Health, University of Naples Federico II, Via S. Pansini 5, 80133 Naples, Italy

**Keywords:** Vessel density, Radial peripapillary capillary plexus, Preperimetric glaucoma, Mild cognitive impairment, Optical coherence tomography angiography

## Abstract

**Purpose:**

To investigate the vessel density (VD) of the radial peripapillary capillary (RPC) plexus in patients affected by preperimetric glaucoma (PPG), amnestic mild cognitive impairment (aMCI) and in a healthy control group using optical coherence tomography angiography (OCTA) in order to clarify the pathogenetic mechanisms of these neurodegenerative diseases.

**Methods:**

In this prospective study, we studied 54 eyes of 54 patients with PPG, 54 eyes of 54 patients with aMCI and 54 healthy controls. All subjects underwent structural spectral domain optical coherence tomography (SD)-OCT to assess the ganglion cell complex (GCC) and the retinal nerve fibre layer (RNFL). OCTA was used to evaluate the VD of the RPC in different regions (whole image, inside disc and peripapillary).

**Results:**

The PPG and aMCI groups showed a statistically significant reduction in SD-OCT and parameters with respect to controls (*p* < 0.001). No statistically significant difference was found in GCC and RNFL parameters between the two study groups (*p* > 0.05). At OCTA examination, PPG and aMCI patients exhibited a statistically significant reduction in the VD of the RPC in whole image, inside and peripapillary regions compared to healthy controls (*p* < 0.001). When comparing the two study groups, the OCTA parameters were significantly impaired in PPG with respect to aMCI patients. Significant correlations were found between structural OCT and OCTA parameters in PPG and aMCI groups (*p* < 0.05).

**Conclusions:**

RPC vessel density could represent a helpful and sensible biomarker to identify early retinal microvascular changes in PPG and MCI in order to better understand the vascular pathophysiological mechanisms involved in these neurodegenerative diseases.
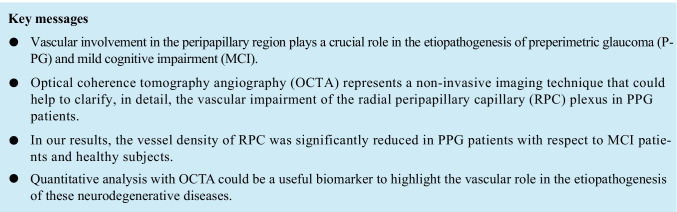

## Introduction

Primary open-angle glaucoma (POAG) is an optic neuropathy that causes progressive damage to neuroretinal structures (ganglion cell complex and retinal nerve fibre layer) as well as visual field loss [1].

The prevalence of POAG increases with age, and the main pathogenetic mechanisms are increased intraocular pressure (IOP) and peripapillary microvascular impairment [2–4].

Preperimetric glaucoma (PPG) represents a stage characterised by the presence of early structural and blood flow damage to the optic disc before the appearance of visual field defects [5], confirming the importance of the vascular role in the pathophysiological mechanisms of this disease.

Moreover, several studies have demonstrated an increased prevalence of alterations in the central nervous system (CNS) in glaucomatous patients, suggesting that POAG could be a neurodegenerative disease [6, 7].

Alzheimer’s disease (AD) is a progressive neurodegenerative disorder, causing dementia in the elderly. It is characterised by cerebral vascular damage [8, 9]. The ganglion cell complex (GCC) and retinal nerve fibre layer (RNFL) loss in these patients have been suggested to be potential biomarkers of neuronal impairment in AD [10].

Mild cognitive impairment (MCI), in particular, is a condition of cognitive decline that does not completely compromise daily functions [11, 12]. It features a significant loss of retinal blood flow before the appearance of structural damages, demonstrating the vascular role as an early and useful biomarker in this neurodegenerative disorder [13]. Since the retinal and cerebral small vessels show similar anatomical features, the identification of retinal vascular changes in the early stages of glaucoma and AD (namely, PPG and MCI, respectively) could shed light on the pathogenesis of these diseases [14, 15].

Previous studies have focused on early blood flow changes in the optic nerve head in PPG and MCI [16, 17] without comparing the degree of retinal vascular impairment between these diseases.

The aim of this observational study was to evaluate the microvascular features of the papillary region using optical coherence tomography angiography (OCTA) in patients affected by PPG and MCI in order to better understand the pathophysiological mechanisms underlying these neurodegenerative disorders.

## Methods

In this observational study, 162 eyes from 162 patients were studied from January 2019 to January 2020 at the University of Naples “Federico II”.

Fifty-four eyes of 54 patients included in the study were affected by preperimetric glaucoma (PPG), 54 eyes of 54 patients showed amnestic mild cognitive impairment (aMCI) and 54 eyes of 54 healthy subjects represented the control group.

All patients and controls underwent a complete ophthalmological examination, including the best-corrected visual acuity (BCVA) evaluation, intraocular pressure (IOP) with Goldmann applanation tonometry, biomicroscopy, gonioscopy, central corneal thickness, fundus examination, visual field (VF), spectral domain-optical coherence tomography (SD-OCT) and OCT angiography (OCTA).

Moreover, an extensive neuropsychological battery, including the mini-mental state evaluation (MMSE), was performed by neurologists to examine each subject for aMCI diagnosis [11, 18, 19].

PPG group presented optic nerve head changes such as localised rim thinning, increased cupping and inter-eye cup asymmetry > 0.2. Moreover, VF showed normal glaucoma hemifield test results, normal mean deviation (MD) and pattern standard deviation (PSD) [20].

All PPG patients underwent topical medication with beta blockers and showed values of IOP within the normal range.

The group affected by aMCI scored ≤ 27 points on MMSE and exhibited a preservation of ability to complete daily life activities.

Healthy controls showed no family history of glaucoma or other ocular disease, IOP less than 21 mmHg, normal visual field test, SD-OCT parameters within normal limits, a normal score on MMSE (≥ 27 points) and no cognitive deficits at neuropsychological testing.

Exclusion criteria included the presence of congenital eye disorders, myopia greater than 6 diopters, history of ocular surgery, vitreoretinal disease, uveitis, diabetic retinopathy, presence of significant lens opacities, history of other neurological or psychiatric disorders and low-quality images obtained with OCT.

Patients with a history of stroke, hypertension, diabetes, depression and neurodegenerative disorders other than Alzheimer disease were also excluded.

During enrollment, all patients underwent systemic blood pressure measurements that turned to be within the normal range.

The study was approved by the Institutional Review Board of the University of Naples “Federico II” (protocol number: 142/19), and all investigations adhered to the tenets of the Declaration of Helsinki. Signed informed consent was obtained from each subject.

### Visual field

Visual field was analysed by perimetry Humphrey field analyzer with Swedish interactive thresholding algorithm (SITA) standard 30–2 test program (Carl Zeiss Meditec, Dublin, CA, USA). Only reliable tests that showed fixation losses less than 20%, false-positive and false-negative errors less than 15%, were considered. The perimeter software also calculated MD and PSD.

### Spectral domain optical coherence tomography

Mean circumpapillary RNFL and GCC thickness were evaluated after pupillary dilation by SD-OCT (software RTVue XR version 2017.1.0.151, Optovue Inc., Fremont, CA, USA). The optic nerve head map protocol evaluated the circumpapillary RNFL on measurements obtained around a circle 3.45 mm in diameter centred on the optic disc.

The GCC thickness was measured from the internal limiting membrane to the outer boundary of the inner plexiform layer on a squared grid (7 mm × 7 mm) on the central macula, centred 1 mm temporal to the fovea [21]. Only high-quality images, as defined by a signal strength index above 40, were accepted. The examiner rejected scans that had motion artefacts, poor centration, incorrect segmentation or poor focus.

### OCT angiography

The XR Avanti AngioVue OCTA (software ReVue version 2017.1.0.151, Optovue Inc., Fremont, CA, USA) is a device based on a split-spectrum amplitude de-correlation algorithm (SSADA) [22].

The AngioVue disc mode automatically segmented the vessel density (VD) of the radial peripapillary capillary (RPC) analysing the superficial retinal layers from the inner limiting membrane (ILM) to the RNFL posterior boundary. The VD was defined as the percentage of the peripapillary region occupied by blood vessels [23].

VD measurements of the RPC were automatically obtained for the entire scanned area, the area inside the optic disc and the peripapillary region. The whole image was evaluated over an area scan of 4.5 × 4.5 mm centred on the optic disc. Inside disc refers to the area inside an ellipse fitted to the optic disc boundary. Peripapillary region is measured in a 0.75-mm-wide elliptical annulus extending outward from the optic disc boundary [23].

OCT and OCTA protocols were performed by the examiner that did not know the study group analysed. Images with a signal strength index less than 40, residual motion artefacts, incorrect segmentation and low centration and focus were excluded from each analysis.

### Statistical analysis

Statistical analysis was performed with the Statistical Package for Social Sciences (Version 20.0 for Windows; SPSS Inc., Chicago, Ill, USA). The chi-squared test was used to determine differences in terms of sex. One-way analysis of variance (ANOVA) followed by Bonferroni post hoc analysis was used to evaluate separately differences for each OCT and OCTA parameter among the study groups.

The relationship between the OCT and OCTA parameters in glaucoma and aMCI groups was evaluated by Pearson’s correlation. A *p*-value < 0.05 was considered statistically significant.

## Results

A total of 162 eyes from 162 subjects, of whom 54 were PPG patients (26 females, 28 males, mean age 72.29 ± 7.05 years), 54 were aMCI patients (30 females, 24 males, mean age 73 ± 6.6 years) and 54 were healthy subjects (29 females, 25 males, mean age 72.66 ± 7.05 years), were included in this observational study. There were no statistically significant differences in age (*p* = 0.859) and sex (*p* = 0.380) among the groups. The MMSE score was significantly lower in the aMCI group with respect to other groups (*p* < 0.001). Mean IOP did not differ among the study groups (*p* = 0.111) (Table 1).

**Table 1:** Demographic and clinical characteristics of controls, preperimetric glaucoma patients and amnestic mild cognitive impairment

	Controls	PPG	aMCI	Anova
				*p*
**Eyes (*n*)**	54	54	54	
**Age (years)**	72.66 ± 7.05	72.29 ± 7.05	73 ± 6.6	0.859
**Sex (female/male)**	29/25	26/28	30/24	0.380†
**BCVA (logMAR)**	0.09 ± 0.09	0.11 ± 0.09	0.1 ± 0.09	0.604
**IOP (mmHg)**	14.34 ± 1.84	15.13 ± 1.95	14.72 ± 2.05	0.111
**MD (dB)**	− 0.51 ± 1.17	− 0.3 ± 1.4	0.19 ± 1.24	0.763
**PSD (dB)**	2.21 ± 0.38	2.14 ± 0.72	2.04 ± 0.55	0.829
**MMSE (points)**	28.42 ± 1.27	28.20 ± 1.40	26.51 ± 1.8	< 0.001

Data are expressed as mean ± SD. *PPG*, preperimetric glaucoma; *aMCI*, amnestic mild cognitive impairment; *BCVA*, best-corrected visual acuity; *logMAR*, logarithm of the minimum angle of resolution; *IOP*, intraocular pressure; *MD*, mean deviation; *PSD*, pattern standard deviation; *dB*, decibel; *MMSE*, mini-mental state examination. One-way analysis of variance (ANOVA) followed by Bonferroni post hoc analysis, †chi-squared test. Statistical significance *P*-value < 0.05

As shown in Table 2, the structural SD-OCT for PPG patients showed a statistically significant reduction in GCC parameters with respect to controls (*p* < 0.001) as well as in RNFL parameters (*p* < 0.001).

**Table 2:** Differences in each structural SD-OCT among three study groups (controls, amnestic mild cognitive impairment and preperimetric glaucoma patients)

SD-OCT (µm)	Control group	PPG	aMCI	Anova	*P*-value for pair comparison
				*P*-value	
**GCC average**	98.59 ± 6.61	90.16 ± 6.01	91.72 ± 9.46	< 0.001	▲ < 0.001
					◊ < 0.001
					●0.851
**GCC superior**	99.62 ± 6.53	91.75 ± 7.31	92.38 ± 9.68	< 0.001	▲ < 0.001
					◊ < 0.001
					●0.978
**GCC inferior**	96.38 ± 7.56	88.62 ± 7.19	91.44 ± 9.23	0.001	▲ < 0.001
					◊0.042
					●0.477
**RNFL average**	102.03 ± 9.32	94.55 ± 9.09	96.42 ± 8.58	< 0.001	▲ < 0.001
					◊0.004
					●0.846
**RNFL superior**	104.83 ± 9.44	96.66 ± 9.59	97.75 ± 9 83	< 0.001	▲ < 0.001
					◊0.001
					●0.965
**RNFL inferior**	100.55 ± 10.19	92.64 ± 9 30	95.38 ± 8.72	< 0.001	▲ < 0.001
					◊0.015
					●0.399

Data are expressed as mean ± SD. *GCC*, ganglion cell complex; *RNFL*, retinal nerve fibre layer; *PPG*, preperimetric glaucoma; *aMCI*, amnestic mild cognitive impairment. ▲Control vs PPG, ◊control vs aMCI, ●PPG vs aMCI. One-way analysis of variance (ANOVA) followed by Bonferroni post hoc analysis. Statistical significance *P*-value < 0.05

A statistically significant reduction in SD-OCT parameters was also found in aMCI patients compared to controls (*p* < 0.001, *p* = 0.004 for GCC average and RNFL average, respectively).

When comparing aMCI and PPG groups, there were no statistically significant differences in GCC and RNFL parameters (*p* > 0.05).

At OCTA examination, PPG patients exhibited a statistically significant decrease in the VD of RPC with respect to controls in whole image, peripapillary region and inside disc (*p* < 0.001). Different behaviours were found in the VD of RPC in aMCI eyes that revealed a reduction compared to controls, although it was not significantly reduced in each sector (*p* > 0.05).

**Fig. 1:**
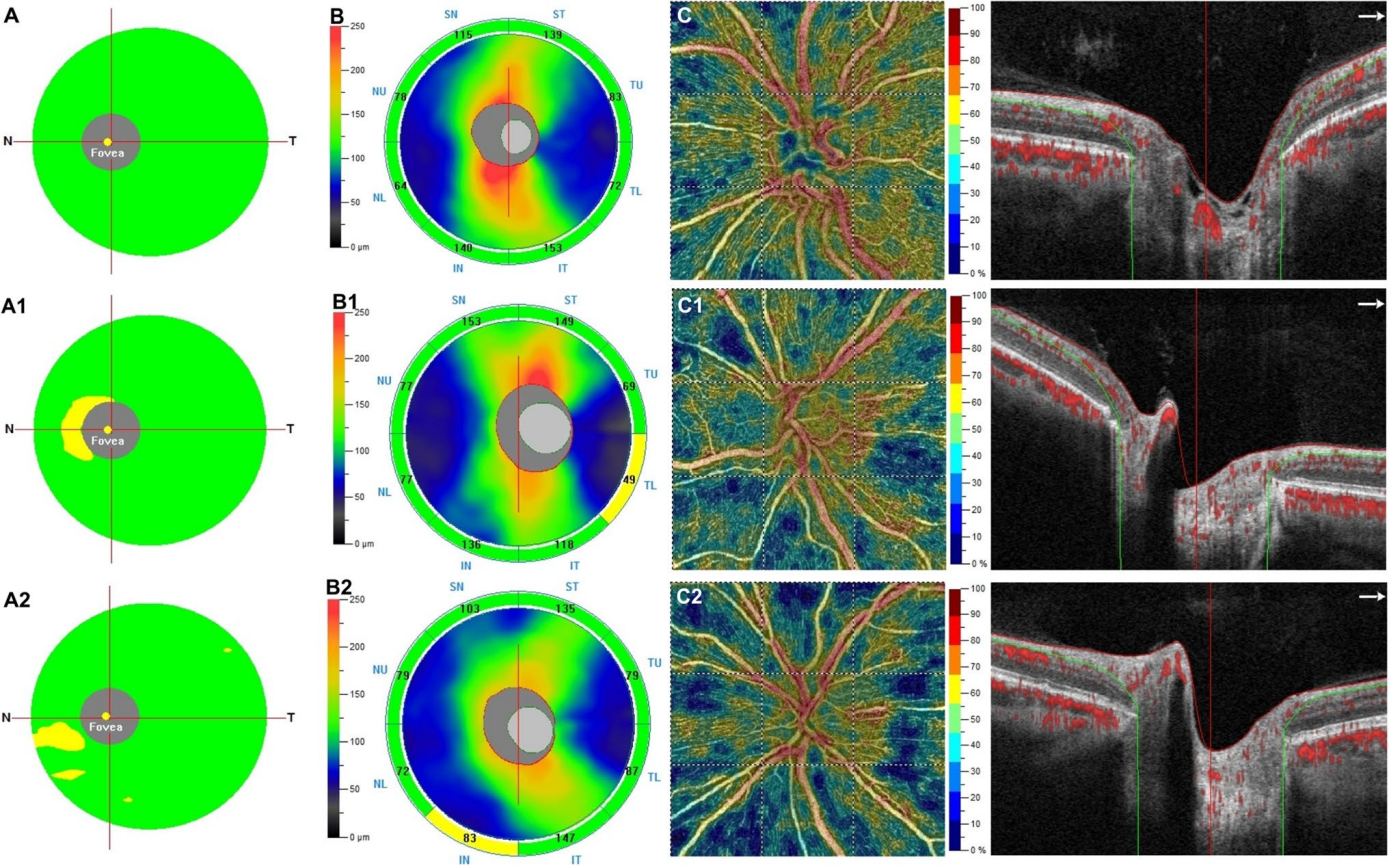
Top row: left eye of a 63-year-old female healthy subject shows normal thickness in ganglion cell complex (GCC) (**A**) and in retinal nerve fibre layer (RNFL) (**B**) at structural OCT (SD-OCT). Optical coherence tomography angiography (OCTA) reveals normal vessel density in the radial peripapillary capillary (RPC) plexus (**C**). Second row: left eye of a 65-year-old male patient with amnestic mild cognitive impairment (aMCI) reveals focal reduction in GCC and RNFL at SD-OCT (A1, B1) and in vessel density of the RPC plexus (C1) at OCTA with respect to the healthy subject. Bottom row: left eye of a 66-year-old female patient with preperimetric glaucoma shows focal reduction in GCC (A2) and RNFL (B2). On OCTA examination, the vessel density of the RPC plexus (C2) was revealed to be decreased with respect to the aMCI subject and healthy control

The PPG group revealed a statistically significant reduction in the VD of RPC compared to the aMCI group in whole image (*p* = 0.008), peripapillary region (*p* = 0.005) and in inside disc (*p* = 0.007) (Table 3, Fig. 1).

**Table 3:** Differences in each OCT angiography vessel density among three study groups (controls, amnestic mild cognitive impairment and preperimetric glaucoma patients)

RPC VD (%)	Control group	PPG	aMCI	Anova	*P*-value for pair comparison
				*P*-value	
**Whole image**	54.39 ± 3.14	50.07 ± 5.02	52.52 ± 4.11	< 0.001	▲ < 0.001
					◊ 0.125
					●0.008
**Inside disc**	50.88 ± 3.05	44.11 ± 3.50	48.41 ± 3.34	< 0.001	▲ < 0.001
					◊0.336
					●0.007
**Peripapillary region**	51.44 ± 4.65	47.32 ± 4.13	49.77 ± 4.44	< 0.001	▲ < 0.001
					◊0.296
					●0.005

Data are expressed as mean ± SD. *RPC VD*, radial peripapillary capillary plexus vessel density; *PPG*, preperimetric glaucoma; *aMCI*, amnestic mild cognitive impairment. ▲Control vs PPG, ◊control vs aMCI, ●PPG vs aMCI. One-way analysis of variance (ANOVA) followed by Bonferroni post hoc analysis. Statistical significance *P*-value < 0.05

Finally, we evaluated the relationships between the structural SD-OCT and OCTA parameters in PPG and aMCI patients. We found a significant correlation between GCC and RNFL average and the VD of RPC (whole image and peripapillary region) in both groups (*p* < 0.05) (Table 4).

**Table 4:** Pearson correlation coefficient between SD-OCT and OCTA parameters in amnestic mild cognitive impairment and preperimetric glaucoma patients

	PPG		aMCI	
	*r*	*p*	*r*	*p*
**GCC — whole VD**	0.435	0.015	0.268	0.042
**GCC — inside disc VD**	0.047	0.523	0.028	0.840
**GCC — peripapillary VD**	0.371	0.038	0.193	0.043
**RNFL — whole VD**	0.351	0.027	0.337	0.013
**RNFL — inside disc VD**	0.128	0.651	0.047	0.737
**RNFL — peripapillary VD**	0.372	0.012	0.325	0.016

*RPC VD*, radial peripapillary capillary plexus vessel density; *PPG*, preperimetric glaucoma; *aMCI*, amnestic mild cognitive impairment;

*R*, Pearson’s correlation coefficient. Statistical significance *P* < 0.05

## Discussion

This study is the first to compare the structural and microvascular changes in the papillary region between PPG and aMCI patients in order to better understand the pathophysiological mechanisms of these neurodegenerative diseases.

Our results demonstrated a significant GCC and RNFL loss in both study groups with respect to controls, as confirmed by previous studies [9, 10], while no significant difference was found in the comparison between two groups, supporting the hypothesis that PPG and aMCI could be involved in similar neurodegenerative processes [15, 24, 25].

Several studies have hypothesised that the reduced cerebrospinal fluid (CSF) pressure, common to both disease, could be involved in neurodegenerative processes [26]. Indeed the cerebral choroid plexus, which is responsible of the production of CSF within cerebral ventricles, would determine a reduction in CSF when undergoing ageing [27, 28].

In glaucomatous patients, the low CSF pressure, together with an increased IOP, would cause high translaminar pressure [29–32]. Brain atrophy in AD patients would similarly decrease the secretion of CSF [27]. These mechanisms, determining a reduction in optic nerve subarachnoid space with lamina cribrosa damage of the optic disc [25, 26, 33], are crucial in the pathogenesis and progression of these disorders.

Previous studies reported in MCI the role of the cerebral amyloid angiopathy in the hypoperfusion. β-amyloid deposition around retinal vascular walls may damage endothelial cells reducing the vascular lumen and then the vascular perfusion [34, 35].

Also the glaucomatous pathogenesis supported a vascular theory according to which this optic neuropathy is a consequence of reduced ocular blood flow due to vascular dysregulation, local vasospasm, hypertension and nocturnal hypotension that lead to axonal ischemia [4, 36–38].

Moreover, the increased IOP would influence the optic nerve perfusion, determining direct effects on laminar microvascular occlusion and indirect effects decreasing the diffusion of axonal nutrients [39].

Our results confirmed the reduced vessel density in papillary region in PPG and MCI by OCTA with respect to controls, as reported by previous studies [13, 40–43].

Regarding the correlation studies, we found a significant relationship between reduced papillary blood flow and retinal structural loss in PPG and MCI, which can be explained by the fact the RPC plexus is located in peripapillary RNFL and contributes to the metabolic demand of the ganglion cells and retinal axonal layers [44]. Therefore, the retinal structural loss, occurring in these disorders, with concomitant reduced metabolic demand would determine the impaired papillary blood flow.

Interesting results have been found in the comparison between two study groups that showed a significant loss in VD of the RPC in PPG with respect to aMCI.

These findings would demonstrate the retinal vascular component loss in the pathogenesis of glaucoma disease, confirming the vascular theory [45, 46].

In conclusion, PPG and MCI are two neurodegenerative diseases with different clinical features presenting a retinal vascular reduction with respect to control. This VD impairment was more evident in glaucoma disease respect to MCI. RPC vessel density could be a helpful and sensible biomarker for identifying early retinal microvascular changes in order to better understand the pathophysiological mechanisms involved in these neurodegenerative diseases.

This study has several limitations including the relatively small sample size of each group and the single-blind design of the study. Further longitudinal studies with larger sample sizes are required to obtain more evidence on usefulness of OCTA in following up and to compare the retinal vascular changes in these neurodegenerative disorders.

## References

[1] Weinreb RN, Khaw PT (2004). Primary open-angle glaucoma. Lancet Lond Engl.

[2] Nickells RW, Howell GR, Soto I, John SW (2012). Under pressure: cellular and molecular responses during glaucoma, a common neurodegeneration with axonopathy. Annu Rev Neurosci.

[3] Yanagi M, Kawasaki R, Wang JJ, Wong TY, Crowston J, Kiuchi Y (2011). Vascular risk factors in glaucoma: a review. Clin Exp Ophthalmol.

[4] Flammer J, Orgül S, Costa VP, Orzalesi N, Krieglstein GK, Serra LM, Renard JP, Stefánsson E (2002). The impact of ocular blood flow in glaucoma. Prog Retin Eye Res.

[5] Cennamo G, Montorio D, Velotti N, Sparnelli F, Reibaldi M, Cennamo G (2017). Optical coherence tomography angiography in pre-perimetric open-angle glaucoma. Graefes Arch Clin Exp Ophthalmol.

[6] Jain S, Aref AA (2015). Senile dementia and glaucoma: evidence for a common link. J Ophthalmic Vis Res.

[7] Gupta N, Ang LC, Noel de Tilly L, Bidaisee L, Yücel YH (2006). Human glaucoma and neural degeneration in intracranial optic nerve, lateral geniculate nucleus, and visual cortex. Br J Ophthalmol.

[8] Alzheimer’s Association. (2015) Alzheimer’s disease facts and figures. Alzheimers Dement 11:332–84. 10.1016/j.jalz.2015.02.003 25984581

[9] Budson AE, Solomon PR (2012). New diagnostic criteria for Alzheimer’s disease and mild cognitive impairment for the practical neurologist. Pract Neurol.

[10] Garcia-Martin E, Bambo MP, Marques ML, Satue M, Otin S, Larrosa JM, Polo V, Pablo LE (2016). Ganglion cell layer measurements correlate with disease severity in patients with Alzheimer’s disease. Acta Ophthalmol.

[11] Burns A, Zaudig M (2002). Mild cognitive impairment in older people. Lancet.

[12] Mitchell AJ, Shiri-Feshki M (2009). Rate of progression of mild cognitive impairment to dementia–meta-analysis of 41 robust inception cohort studies. Acta Psychiatr Scand.

[13] Criscuolo C, Cennamo G, Montorio D, Carotenuto A, Strianese A, Salvatore E, Tranfa F, Cennamo G, Lanzillo R, Brescia Morra V (2020). Assessment of retinal vascular network in amnestic mild cognitive impairment by optical coherence tomography angiography. PLoS One.

[14] Patton N, Aslam T, MacGillivray T, Pattie A, Deary IJ, Dhillon B (2005). Retinal vascular image analysis as a potential screening tool for cerebrovascular disease: a rationale based on homology between cerebral and retinal microvasculatures. J Anat.

[15] London A, Benhar I, Schwartz M (2013). The retina as a window to the brain-from eye research to CNS disorders. Nat Rev Neurol.

[16] Kim SB, Lee EJ, Han JC, Kee C (2017). Comparison of peripapillary vessel density between preperimetric and perimetric glaucoma evaluated by OCT-angiography. PLoS One.

[17] Zhang YS, Zhou N, Knoll BM, Samra S, Ward MR, Weintraub S, Fawzi AA (2019). Parafoveal vessel loss and correlation between peripapillary vessel density and cognitive performance in amnestic mild cognitive impairment and early Alzheimer’s disease on optical coherence tomography angiography. PLoS One.

[18] McKhann GM, Knopman DS, Chertkow H (2011). The diagnosis of dementia due to Alzheimer’s disease: recommendations from the national institute on aging–Alzheimer’s Association workgroups on diagnostic guidelines for Alzheimer’s disease. Alzheimers Dement.

[19] Folstein MF, Folstein SE, McHugh PR (1975). “Mini-mental state”. A practical method for grading the cognitive state of patients for the clinician. J Psychiatr Res.

[20] Tan O, Chopra V, Lu AT, Schuman JS, Ishikawa H, Wollstein G, Varma R, Huang D (2009). Detection of macular ganglion cell loss in glaucoma by Fourier-domain optical coherence tomography. Ophthalmology.

[21] Hirashima T, Hangai M, Nukada M, Nakano N, Morooka S, Akagi T, Nonaka A, Yoshimura N (2013). Frequency-doubling technology and retinal measurements with spectral-domain optical coherence tomography in preperimetric glaucoma. Graefes Arch Clin Exp Ophthalmol.

[22] Jia Y, Tan O, Tokayer J, Potsaid B, Wang Y, Liu JJ, Kraus MF, Subhash H, Fujimoto JG, Hornegger J, Huang D (2012). Split spectrum amplitude-decorrelation angiography with optical coherence tomography. Opt Express.

[23] Rao HL, Pradhan ZS, Weinreb RN, Reddy HB, Riyazuddin M, Dasari S, Palakurthy M, Puttaiah NK, Rao DAS, Webers CAB (2016). Regional comparisons of optical coherence tomography angiography vessel density in primary open-angle glaucoma. Am J Ophthalmol.

[24] Jones-Odeh E, Hammond CJ. (2015): How strong is the relationship between glaucoma, the retinal nerve fibre layer, and neurodegenerative diseases such as Alzheimer’s disease and multiple sclerosis? Eye(Lond):1270–84. 10.1038/eye.2015.158 PMC4815693 26337943

[25] Wostyn P (2019). Glaucoma as a dangerous interplay between ocular fluid and cerebrospinal fluid. Med Hypotheses.

[26] Wostyn P, Audenaert K, De Deyn PP. (2008) Alzheimer’s disease-related changes in diseases characterized by elevation of intracranial or intraocular pressure. Clinical Neurology and Neurosurgery 110: 101–109. 10.1016/j.clineuro.2007.10.011 18061341

[27] Serot JM, Bene MC, Faure GC (2006). Choroid plexus, aging of the brain, and Alzheimer’s disease. Front Biosci..

[28] May C, Kaye JA, Atack JR, Schapiro MB, Friedland RP, Rapoport SI (1990). Cerebrospinal fluid production is reduced in healthy aging. Neurology.

[29] Berdahl JP, Allingham RR (2010). Intracranial pressure and glaucoma. Curr Opin Ophthalmol.

[30] Morgan WH, Balaratnasingam C, Cringle SJ, Yu DY (2008). Glaucoma and cerebrospinal fluid pressure. Ophthalmology.

[31] Berdahl JP, Allingham RR, Johnson DH (2008). Cerebrospinal fluid pressure is decreased in primary open-angle glaucoma. Ophthalmology.

[32] Cennamo G, Montorio D, Breve MA, Brescia Morra V, Menna F, Cennamo G (2018). Evaluation of optic nerve subarachnoid space in primary open angle glaucoma using ultrasound examination. PLoS One.

[33] Wostyn P, Audenaert K, De Deyn PP. (2008) An abnormal high trans-lamina cribrosa pressure difference: a missing link between Alzheimer’s disease and glaucoma? Clinical Neurology and Neurosurgery 110: 753–754. 10.1016/j.clineuro.2008.05.019 18603354

[34] de la Torre JC (2013). Vascular risk factors: a ticking time bomb to Alzheimer’s disease. Am J Alzheimers Dis Other Demen.

[35] William R. Brown, Clara R. Thore. Review (2011) Cerebral microvascular pathology in aging and neurodegeneration. Neuropathol Appl Neurobiol 37: 56–74. 10.1111/j.1365-2990.2010.01139.x PMC3020267 20946471

[36] Bonomi L, Marchini G, Marraffa M, Bernardi P, Morbio R, Varotto A (2000). Vascular risk factors for primary open angle glaucoma: the Egna-Neumarkt study. Ophthalmology.

[37] Harris A, Kagemann L, Ehrlich R, Rospigliosi C, Moore D, Siesky B (2008). Measuring and interpreting ocular blood flow and metabolism in glaucoma. Can J Ophthalmol.

[38] Resch H, Garhofer G, Fuchsjäger-Mayrl G, Hommer A, Schmetterer L (2009). Endothelial dysfunction in glaucoma. Acta Ophthalmol.

[39] Burgoyne CF, Downs JC, Bellezza AJ, Suh JK, Hart RT (2005). The optic nerve head as a biomechanical structure: a new paradigm for understanding the role of IOP-related stress and strain in the pathophysiology of glaucomatous optic nerve head damage. Prog Retin Eye Res.

[40] Kim SB, Lee EJ, Han JC, Kee C (2017). Comparison of peripapillary vessel density between preperimetric and perimetric glaucoma evaluated by OCT-angiography. PLoS One.

[41] Mangouritsas G, Koutropoulou N, Ragkousis A, Boutouri E, Diagourtas A (2019). Peripapillary vessel density in unilateral preperimetric glaucoma. Clin Ophthalmol.

[42] Akil H, Huang AS, Francis BA, Sadda SR, Chopra V (2017). Retinal vessel density from optical coherence tomography angiography to differentiate early glaucoma, pre-perimetric glaucoma and normal eyes. PLoS One.

[43] Kumar RS, Anegondi N, Chandapura RS, Sudhakaran S, Kadambi SV, Rao HL, Aung T, Sinha RA (2016). Discriminant function of optical coherence tomography angiography to determine disease severity in glaucoma. Invest Ophthalmol Vis Sci.

[44] Campbell JP, Zhang M, Hwang TS, Bailey ST, Wilson DJ, Jia Y, Huang D (2017). Detailed vascular anatomy of the human retina by projection-resolved optical coherence tomography angiography. Sci Rep.

[45] Emre M, Orgül S, Gugleta K, Flammer J (2004). Ocular blood flow alteration in glaucoma is related to systemic vascular dysregulation. Br J Ophthalmol.

[46] Schmidl D, Garhofer G, Schmetterer L (2011). The complex interaction between ocular perfusion pressure and ocular blood flow-relevance for glaucoma. Exp Eye Res.

